# Metapopulation patterns of additive and nonadditive genetic variance in the sea bass (*Dicentrarchus labrax*)

**DOI:** 10.1002/ece3.2832

**Published:** 2017-03-21

**Authors:** Bruno Guinand, Marc Vandeputte, Mathilde Dupont‐Nivet, Alain Vergnet, Pierrick Haffray, Hervé Chavanne, Béatrice Chatain

**Affiliations:** ^1^ Département Biologie‐EcologieUniversité de MontpellierMontpellierFrance; ^2^ UMR CNRS IRD EPHE UM Institut des Sciences de l'Evolution de MontpellierMontpellierFrance; ^3^ INRAUMR1313 GABIDomaine de VilvertJouy‐en‐JosasFrance; ^4^ IfremerUMR 9190Marine Biodiversity, Exploitation and ConservationPalavas‐les‐FlotsFrance; ^5^ SYSAAFCampus de BeaulieuRennesFrance; ^6^ Istituto Sperimentale Lazzaro SpallanzaniRivolta d'AddaItaly

**Keywords:** additive and nonadditive variance, Darwin's corollary, heterosis, mito‐nuclear epistasis, pleiotropy

## Abstract

Describing and explaining the geographic within‐species variation in phenotypes (“phenogeography”) in the sea over a species distribution range is central to our understanding of a variety of eco‐evolutionary topics. However, phenogeographic studies that have a large potential to investigate adaptive variation are overcome by phylogeographic studies, still mainly focusing on neutral markers. How genotypic and phenotypic data could covary over large geographic scales remains poorly understood in marine species. We crossed 75 noninbred sires (five origins) and 26 dams (two origins; each side of a hybrid zone) in a factorial diallel cross in order to investigate geographic variation for early survival and sex ratio in the metapopulation of the European sea bass (*Dicentrarchus labrax*), a highly prized marine fish species. Full‐sib families (*N *= 1,950) were produced and reared in a common environment. Parentage assignment of 7,200 individuals was performed with seven microsatellite markers. Generalized linear models showed significant additive effects for both traits and pleiotropy between traits. A significant nonadditive genetic effect was detected. Different expression of traits and distinct relative performances were found for reciprocal crosses involving populations located on each side of the main hybrid zone located at the Almeria‐Oran front, illustrating asymmetric reproductive isolation. The poor fitness performance observed for the Western Mediterranean population of sea bass is discussed as it represents the main source of seed hatchery production, but also because it potentially illustrates nonadaptive introgression and maladaptation.

## 1 Introduction

As natural selection acts primarily on phenotypes, describing the geographic within‐species variation in phenotype in the sea is important to a better understanding of a variety of ecological and evolutionary topics (Conover, Clarke, Munch, & Wagner, 2006; Sotka, 2012) and to management of marine species (Marshall, Monro, Bode, Keough, & Swearer, 2010; Swain & Foote, 1999). Conover et al. (2006) coined the term “phenogeography” to study the distribution and the genetic basis of phenotypic variation in nature as opposed to phylogeography (i.e., the geography of phenotypic variation *vs* the geography of lineages). The integration of phenogeography and phylogeography should contribute to better understanding of functional phenotypic evolution, and then fitness performance within and among lineages (Zamudio, Bell, & Mason, 2016). However, phenogeography—with the noticeable exception of counter‐ and cogradient variation studies (Conover, Duffy, & Hice, 2009; Hice, Duffy, Munch, & Conover, 2012) and few Q_ST_‐F_ST_ studies (DeFaveri & Merilä, 2013)—remains largely neglected in marine species. Indeed, while studies regarding changes in quantitative phenotypic differences mediated by trait plasticity and/or genetic processes are obviously present in the literature associated to evolutionary ecology of marine species (Conover et al., 2006; Sanford & Kelly, 2011), most studies concentrated at the molecular level (i.e., gene expression studies, population genetics/genomics) rather than on quantitative biological traits aiming to document the causes of variation of an organism phenotype over a large geographic scale, and their impact on fitness.

The genetic differentiation between Mediterranean and Atlantic populations of plant and animal marine species was reviewed by Patarnello, Volckaert, and Castilho (2007), showing they are often structured as two divergent lineages. However, with few exceptions (e.g., Yebra, Bonnet, Harris, Lindeque, & Peijnenburg, 2011), no geographic study of phenotypic or life‐history variation was carried out at this large scale. Consequently, how the genotypic and phenotypic data are both structured and could covary over large geographic scales is very poorly assessed. We guess a similar observation is true for other marine areas worldwide, despite marine species provide good opportunity for “phenogeographic” studies. Indeed, they often exhibit shallow population structure, with large panmictic populations separated by a few well‐identified genetic breaks mostly due to secondary contact zones established after populations evolved in isolation (Hellberg, 2009; Hellberg, Burton, Neigel, & Palumbi, 2002). Thus, it is possible to sample most if not all relevant populations over a species distribution area and to investigate trait variation and performance at the scale of a whole metapopulation.

**Figure 1:**
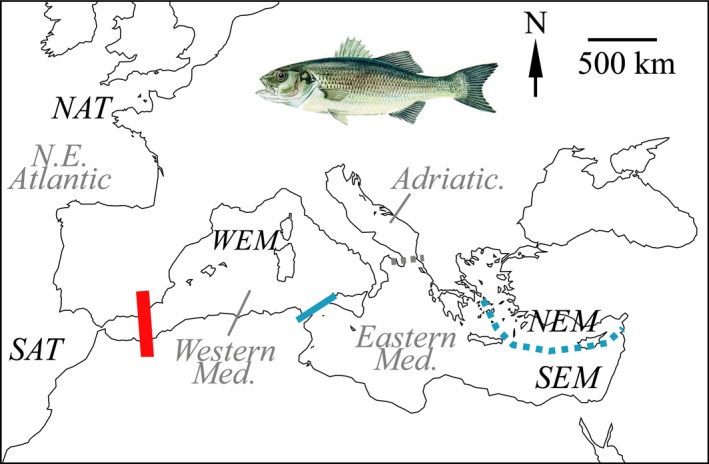
Overview of genetic population structure in the sea bass (*Dicentrarchus labrax*). The red line indicates the Almeria‐Oran front where a secondary contact zone separating the Atlantic and the Mediterranean populations of sea bass is observed. Mitochondrial and nuclear DNA markers show genetic differentiation at this front (e.g., mtDNA: Lemaire et al., 2005; SNPs: Tine et al., 2014). The blue line indicated the Siculo‐Tunisian strait where genetic differentiation between Eastern and Western Mediterranean populations is observed, but only based on nuclear markers. The broken blue line indicates further differentiation of Eastern Mediterranean metapopulation in a northern and a southern component. Based on nuclear markers, this differentiation has been reported twice in the literature (Castilho & Ciftci, 2005; Quéré et al., 2012), but geographic boundaries are poorly known. It is also observed in other species, such like the grey mullet (*Mugil cephalus*; Durand et al., 2013). Fish used in this study originated from main genetic subdivisions and include NAT and SAT in the Northeastern Atlantic, then WEM, NEM, and SEM in the Mediterranean Sea. NAT and SAT are hereby separated, but genetic homogeneity was found to prevail in most studies investigating population genetics of the sea bass within the Atlantic (e.g., Naciri et al., 1999; Souche et al., 2015). Note that a recognized Adriatic genetic subdivision (broken gray line) now recognized for sea bass (Souche et al., 2015) was not included in this study as experiments were performed before description of this population. Labels: NAT: North Atlantic, SAT: South Atlantic, WEM: Western Mediterranean, NEM: Northeastern Mediterranean, SEM: Southeastern Mediterranean. See text for further comments

In European waters, the phylogeography of sea bass (*Dicentrarchus labrax*; Moronidae) has been the subject of numerous studies for approximately 30 years (Chatain & Chavanne, 2009; Quéré et al., 2012 for reviews). As for many other species (Patarnello et al., 2007), populations of sea bass have been shown to be genetically differentiated between the Atlantic and the Mediterranean Sea, and within the Mediterranean Sea itself (Quéré et al., 2012; Souche et al., 2015; Tine et al., 2014) (Figure 1). In sea bass, nuclear and mitochondrial (mtDNA) markers provided different pictures of genetic differentiation. Indeed, nuclear data generally demonstrated genetic homogeneity within the Atlantic, while the Mediterranean Sea is genetically structured, with one single panmictic population within the Western Mediterranean Sea (Naciri, Lemaire, Borsa, & Bonhomme, 1999; Quéré et al., 2012; Souche et al., 2015), and one main metapopulation within the Eastern Mediterranean Sea subdivided in slightly genetically differentiated local populations (Bahri‐Sfar, Lemaire, Ben Hassine, & Bonhomme, 2000; Castilho & Ciftci, 2005; Quéré et al., 2012; Souche et al., 2015). The Atlantic and the Eastern Mediterranean populations acted as refuges during glaciations and their populations are considered being ancestral (Patarnello et al., 2007), which is confirmed by the existence of only two clades of mtDNA (one Atlantic, one Mediterranean; Lemaire, Versini, & Bonhomme, 2005; Rondon, 2011; Coscia, Desmarais, Guinand, & Mariani, 2012). A mismatch is then observed between nuclear and mitochondrial data regarding the number of major metapopulation units in sea bass. How two ancestral populations evolved in three genetically distinct units remains unsolved (Quéré et al., 2012).

Atlantic and Mediterranean populations of sea bass are separated by a main secondary contact hybrid zone occurring at the Almeria‐Oran front recorded for both nuclear and mtDNA markers (Lemaire et al., 2005; Tine et al., 2014) and shared by numerous species (Patarnello et al., 2007). The sea bass hybrid zone is precisely dated to the last glacial retreat, ~11,500 years BP (Tine et al., 2014). Using genome‐wide data, Tine et al. (2014) estimated that about two‐thirds of the sea bass nuclear genome almost freely mixed through this hybrid zone, while the remaining portion should contain the genomic regions responsible for the maintenance of reproductive isolation among populations. Additionally, microsatellite data showed the presence of another hybrid zone located at the Siculo‐Tunisian Strait (Bahri‐Sfar et al., 2000; Quéré et al., 2012), which is also shared by other marine fish species (Durand, Blel, Shen, Koutrakis, & Guinand, 2013; Mejri, Lo Brutto, Ben Hassine, & Arculeo, 2009; Patarnello et al., 2007). In comparison, studies of phenotypic traits have been rarely performed at scales relevant for comparison with sea bass genetic studies (Gorshkov, Gorshkova, Meiri, & Gordin, 2004; Costa et al., 2010; Vandeputte et al., 2014). Costa et al. (2010) and Vandeputte et al. (2014) showed that variation in traits related to fish shape and relevant for aquaculture production (e.g., harvest weight, fillet yield), respectively, had a heritable component in sea bass. This might be indicative of adaptive phenotypic variation and then relevant to phenogeography. However, studies of additional phenotypic traits with better relevance to fitness (e.g., survival) have to be performed yet to better determine how phenogeography and phylogeography interact in sea bass.

In this study, we aim to provide a phenogeographic study associated to a phylogeographic study in sea bass. We used noninbred wild sea bass of Atlantic and Mediterranean origins that cover the range of genetically differentiated natural populations reported for this species (five sire populations carrying the nuclear genomes of most populations identified to date, and two dam populations carrying the two mtDNA genomes) and performed a factorial diallel cross to test for the relationship between genetic distance and observed variation in juvenile survival and sex ratio in F_1_ individuals.

## 2 Materials and methods

### 2.1 Broodstock origin

Wild sea bass used to produce the broodstock for this study was collected from five distinct origins in the Northeastern Atlantic or the Mediterranean Sea recognized as the main subpopulations of wild sea bass (Chatain & Chavanne, 2009; Quéré et al., 2012) (Table 1). Former population genetics studies reported that the English Channel and the Bay of Biscay belong to the same panmictic unit in sea bass (Naciri et al., 1999; Lemaire et al., 2005; Fritsch et al., 2007; Coscia et al., 2012; Souche et al., 2015). Panmixia justified to not include further population subdivision (as dam and/or sire) to the data set. The sea bass population from the Gulf of Lions was also found to be panmictic (Garcia de León, Chikhi, & Bonhomme, 1997; Quéré et al., 2012), and, as for NAT, no further population subdivision as sire or dam was included in the analyses. The distinction between NAT and SAT is motivated by findings by Castilho and McAndrew (1998) and Souche et al. (2015) that reported slight genetic differentiation among populations, while some other studies did not report genetic differentiation in this area (e.g., Naciri et al., 1999). This possible genetic differentiation deserved further attention and NAT and SAT were first treated separately in this study, then merged when necessary (see results). The dam and sire lines used in this study cover most parts of sea bass distribution area (Figure 1). This resulted in two parental origins of females (NAT and WEM) that are representative of the two mtDNA clades of sea bass (e.g., Lemaire et al., 2005), and five origins (NAT, SAT, WEM, NEM, SEM) of males that were used as dam and sire in this study, respectively. Males are representative of the genetically differentiated areas recognized for this species (Atlantic, WEM, SEM, NEM) (Figure 1), and the Atlantic was further separated in two populations (NAT, SAT). However, a full diallel cross was not feasible because of the much greater costs and practical difficulties related to transporting live females (as opposed to cryopreserved sperm used for males; Table 1), maintaining them and making them spawn simultaneously. A piece of fin of each parent (wild) individual used in this study was taken for further DNA extraction and kept in 90% ethanol.

**Table 1:** Samples used in this study. Numbers of females and males are reported. Female broodstocks were maintained at Ifremer facilities (Palavas‐les‐Flots, France). Numbers in bracket indicate randomly chosen individuals from each location that were used as sire or dam in crossing experiments (see Figure 3). DNA analyses for males and subsequent crossings made in this study were based on cryopreserved sperm. All cryopreserved straws were stocked in the sperm cryobank maintained at Ifremer. Sperm was cryopreserved as in Fauvel, Suquet, Dréanno, Zonno, and Menu (1998). Labels as in Figure 1

Population	Number of males per sample	Number of females per sample	Fish origin and sampling dates
NAT	17 (15)	71 (9^b^)	English Channel (France, 2004, 2005)South Brittany, Bay of Biscay (France, 2005)
SAT	16 (15)	–	Rio Mira (Portugal, 2005)
WEM	41 (15)	44 (17)	Perpignan, Palavas‐les Flots; Gulf of Lions (France, 2005)
NEM	30 (15)	–	Antalya (Turkey, 2005)
SEM	21 (15)	–	National Center for Mariculture, Eilat (Israel, 1995)^a^

a Fish that initiated the SEM broodstock were caught in August 1995 by the NCM. One hundred and twenty wild sea bass (yearlings; 30–50 g) were captured along the Egyptian coast of the Mediterranean Sea near Port‐Said, also including fingerlings from a brackish‐water lake located in the estuarine area of the Nile River. The first‐generation (unselected) progeny of these parental fish were used as broodstock for the present experiment.

b Initially 10 females, but one did not produce offspring.

### 2.2 Crossing

On 19 February 2007, 15 NAT and 24 WEM mature dams were injected with luteinizing hormone releasing hormone (LHRHa; 10 μg kg^−1^) to induce reproduction. The egg collection of each dam was carried out 3 days after the injection, and spawns of nine NAT and 17 WEM dams were obtained. Eggs were mixed in equal proportions (150 ml per WEM dam, 250 ml per NAT dam except one with 180 ml) in two batches (NAT and WEM). Each batch of eggs was then divided in 75 aliquots, which were individually fertilized (no intersire competition) with the sperm of 15 sires from each geographic origin to produce a theoretical number of 1,950 families of sea bass (26 dams × 75 sires). Sperm cell quality was checked as described in Ky, Vergnet, Molinari, Fauvel, and Bonhomme (2012) in order to retain sires with good semen quality. The fertilized eggs were mixed by sire (*n *=* *5) × dam (*n *=* *2) origins and incubated in 10 different incubators. After 48 hr of incubation, the live eggs (i.e., floating eggs that are an indicator of fertilization rate; Carrillo, Bromage, Zanuy, Serrano, & Prat, 1989) were all mixed in equal proportions leading to ~2.6 million eggs produced. Survival to 48 hr postfertilization was 58% for the NAT females and 59% for WEM females. At 4 days postfertilization (dpf), ~2 million newly hatched larvae were transferred to the facility of Viveiro Vila Nova (Vila Nova de Milfontes, Portugal) for larval rearing.

### 2.3 Larval rearing

Larvae were reared in one 6 m^3^ tank starting with a subsample of 1.09 million fish on 1 March 2007 (9 dpf). The temperature gradually increased from 16 to 20°C at 40 dpf, then fish were transferred to a 15 m^3^ nursery tank. The fish were fed with *Artemia* starting at 13 dpf, then with commercial pellets afterward. Temperature was kept constant around 20°C (19–21°C) until 111 dpf, when 3,000 randomly selected fish were transported to another pregrowing location (Ardag, Eilat; Israel), while the majority remained in the initial site (Viveiro Vila Nova). Therefore, full‐sib families of sea bass grew in a common environment until 111 dpf in order to minimize early‐life genotype‐by‐environment (G×E) interactions. The range of rearing temperatures used until 111 dpf is known to produce an excess of male sea bass (reviewed in Piferrer, Blázquez, Navarro, & González, 2005), certainly due to interactions between genetic, environmental, and epigenetic factors occurring during early development (Navarro‐Martín et al., 2011; Vandeputte, Dupont‐Nivet, Chavanne, & Chatain, 2007). The fish remaining in Portugal were transferred to a 78 m^3^ tank with natural temperature at 127 dpf (*N *=* *105,767; 9.7% survival). Potential G×E interactions introduced after transfer to each pregrowing locations were tested prior to model use and were not found significant (not reported). Furthermore, maternal effects are weak in sea bass because of lecithotrophic embryos. These effects are restricted to the first weeks of life (<4 weeks postfertilization; Saillant, Chatain, Fostier, Przybyla, & Fauvel, 2001) and not detected significant at older age (Dupont‐Nivet et al., 2008). Accordingly, nonadditive genetic variation due to maternal effects or early‐life G×E interactions was unlikely in this study.

Fish in Israel grew faster due to higher temperature, and at 187 dpf (24 g mean weight) 1,800 of them were randomly chosen, and PIT‐tagged in the muscle, on the left dorsal anterior part of the body. A fin‐clip was taken from each tagged fish for further DNA extraction and kept in 90% ethanol. On 26 September 2007 (216 dpf), the Portugal fish had reached an estimated mean body mass of ~20 g, and 5,400 of them were tagged, measured, and sampled as described before. All fish were reared until sexing was possible at *ca*. 1 year of age at which time fish were euthanized and sexed. All experiments were performed according to the European Union regulations concerning the protection and welfare of animals.

### 2.4 Genetic analyses and parental assignment

The 7,200 PIT‐tagged sea bass juveniles were assigned to their parents (*n *=* *101; 26 dams and 75 sires) with seven microsatellite markers: *Dla016*,* Dla020*,* Dla105*,* Dla116*,* Dla119*,* Lab13*, and *Lab3* (Chistiakov et al., 2004; Ciftci, Castilho, & McAndrew, 2002; Garcia De Léon et al., 1995). Tissues were digested in 100 ml lysis buffer containing 25 ml 0.5 mol/L EDTA (pH = 8), 2.5 ml RNAse (4 mg ml^−1^), and 10 ml proteinase K (20 mg ml^−1^). DNA extraction was performed with the Wizard SV Genomic DNA Purification System kit (Promega), according to manufacturer recommendations. Extracts were stored at −20°C. Amplification was performed in a 20‐μl polymerase chain reaction (PCR) mixture containing 25 ng of genomic DNA, 2.0 μl PCR buffer, 1.2 μl MgCl_2_, 0.4 units Amplitaq Gold (Applied Biosystems), 1.25 mmol/L dNTPs mix (Applied Biosystems), and 10 pmol for each primer. The reverse primers were 5′ end‐labelled with FAM, NED, or VIC fluorochromes. The samples were amplified on a Geneamp^®^ PCR System 9600 (Invitrogen) according to the following protocol: 10‐min initial denaturation at 95°C (hot start) followed by 30 cycles of 1 min at 94°C, 30 s at 55°C, 1 min at 72°C and extension at 72°C for 60 min. The polymorphism was screened on an ABI PRISM^®^ 3100 DNA Analyzer (Life Technologies).

Deviations from Hardy–Weinberg equilibrium for each parental line's sample were investigated using the statistics $\hat{f}$ (Weir & Cockerham, 1984) with GENETIX v4.05 (kimura.univ‐montp2.fr/genetix). The null hypothesis (*f *=* *0) of no significant departure from panmixia was tested by randomly permuting alleles (*n *=* *1,000) from the original matrix of genotypes. Pairwise levels of population differentiation among broodstocks were investigated using $\hat{\theta }$ (Weir & Cockerham, 1984), an estimator of *F*_ST_ also implemented in GENETIX v4.05. Significance of $\hat{\theta }$ was tested by permutation (*n *=* *1,000) and corrected for multiple tests. We attempted to assign individuals of each broodstock (NAT, SAT, WEM, NEM, SEM) to their population of origin using arlequin v3.5 (Excoffier & Lischer, 2010). Offspring pedigree was established by exclusion with the VITASSIGN program based on parent and offspring microsatellite genotypes (Vandeputte, Mauger, & Dupont‐Nivet, 2006).

### 2.5 Biological traits and statistical analyses

Survival of each dam × sire cross was estimated from the representation at tagging (~ 6 months; see “Larval rearing”) of each full‐sib family in the 7,200 genotyped offspring. Sex ratio was determined by visual examination of the gonads of 2,877 genotyped and tagged fish that were sacrificed.

The number of fish per population cross at tagging was used as a surrogate for survival, under the hypothesis of equal initial numbers per population cross, and was tested with a generalized linear model (GLM) to assess the existence of differences linked to population and population cross:

$$Y_{ijk}=\mu +l_{i}+p_{j}+m_{k}+pm_{jk}+\varepsilon_{ijk}$$

where *Y*_*ijk*_ is the number of survivors in the offspring of paternal line *j* and maternal line *k* reared in pregrowing location *i*, μ is the population mean, *l*_*i*_ is the fixed effect of pregrowing location *i* (*i *=* *1 for Israel, 2 for Portugal), *p*_*j*_ is the fixed effect of paternal (sire) line *j* (*j *=* *1–5), *m*_*k*_ is the fixed effect of maternal (dam) line *k* (*j *=* *1, 2), *pm*_*jk*_ is the fixed interaction term between paternal line *j* and maternal line *k* (nonadditive sire × dam effects), and ε_*ijk*_ is the random residual. This model was fitted with the SAS‐GENMOD procedure, with a logarithm link function and a Poisson residual distribution, which is appropriate for count data. Individual sire and dam effects on full‐sib family counts were also tested within each paternal and maternal line crosses. Sex‐ratio differences were tested with the same model where *Y*_*ijk*_ was the proportion of females in the offspring of sire line *j* and dam line *k* reared in pregrowing location *i*. In the case of the sex ratio, logit was used as the link function, with a binomial residual, which is appropriate for binary variables (female or male). The family within line information was available, but it could not be used in the analysis owing to the very high number of potential families (*n *=* *1,950) compared to sample size (*n *=* *2,877) which precluded model convergence. This GLM was also used with *j *=* *2 for sire lines. In this case, sires from the Mediterranean (WEM, NEM, and SEM) and from the Atlantic (NAT, SAT) were grouped before analyses.

The interaction effect estimates derived from the survival and the sex‐ratio GLMs (i.e., *pm*_*.*._, above) were regressed on the pairwise values of $\hat{\theta }$ between the NAT and each of the five paternal lines. NAT was retained as a reference because it was one of the extreme populations in terms of genetic distance (Table S1), and was used as both sire and dam line in the experiment (i.e., SEM could not be tested in the same way as a SEM dam is lacking in the current study). This approach allowed the detection of crosses that produced positive or negative nonadditive genetic effects *sensu lato* as a function of the genetic distance among populations.

## 3 Results

### 3.1 Genetic differentiation among parental lines

No deviation from Hardy–Weinberg equilibrium was observed within each broodstock of noninbred fish that initiated the experiment ($\hat{f}$ ranged from −0.044 to 0.009, NS). The mean genetic differentiation among broodstocks was estimated to $\hat{\theta }$ = 0.038 (*p *<* *.001), with all pairs of broodstocks found significantly differentiated with $\hat{\theta }$ ranging from $\hat{\theta }$ = 0.006 (NS) for the Atlantic NAT‐SAT comparison (the only nonsignificant pairwise comparison found in the data; see Table S1) to $\hat{\theta }$ > 0.05 (*p *<* *.001) for comparisons of SEM with each Atlantic population. Results confirmed the genetic homogeneity within the Atlantic, and that the Atlantic samples were genetically distinct of Mediterranean samples. Relative genetic differentiation observed among Atlantic and Mediterranean populations of sea bass is illustrated in Fig. S1. Individuals were all assigned to their population of origin using arlequin, except for SAT (93%) and NAT (92%) because of above‐mentioned nonsignificant genetic differentiation (mean: 97.03%; 98 of 101 broodstock fish correctly assigned).

### 3.2 Parentage assignment

Seven thousand one hundred twenty fish of the 7,200 fish (98.9%) were assigned to a unique parental pair when using the six or seven microsatellite markers. Among the unassigned fish, 41 (0.6%) did not provide reliable DNA amplification, 35 (0.5%) were assigned to two or more parent pairs, and only 4 (0.05%) were not assigned to any parent pair. Unassigned fish were discarded from the analysis.

### 3.3 Survival and representation of populations at tagging

**Figure 2:**
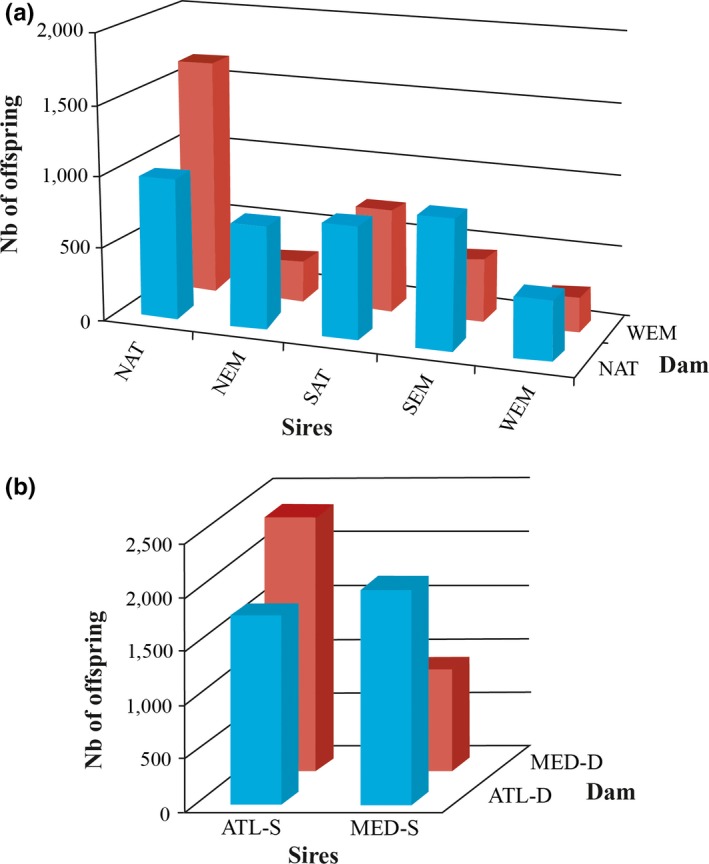
Number of sea bass offspring produced by each cross of (a) initial parental populations at tagging (~6 months), and (b) after groupings Atlantic and Mediterranean sires together. In (a), labels indicate the origin of sea bass populations used as sire (five paternal origins) or dam (two maternal origins). Labels as in Figure 1. In (b): ATL‐D and ATL‐S represent Atlantic dam and sire, respectively, and MED‐D and MED‐S the Mediterranean dam and sire, respectively. ATL‐S was obtained after grouping the NAT and SAT sires; MED‐S after grouping the WEM, NEM, and SEM sires

The representation of parental lines was found to be highly unbalanced at 20 g (step for tagging and DNA collection). This was observed among the sire and the dam lines for both the initial five paternal lines × two maternal lines data set, and for the two paternal lines × two maternal lines data set (i.e., after grouping the Atlantic and Mediterranean lines together; Figure 2). Sex ratio was one exception. The interaction component between sire and dam lines was also found to be highly significant (Table 2). As the number of fish per population cross was standardized at 48 hr postfertilization and larval rearing was performed in common tanks before tagging (i.e., all fish followed a standardized protocol within each of the two pregrowing locations), a differential survival during larval rearing, with both strong additive (significant sire and dam line effects) and nonadditive components certainly occurred (Table 2).

**Table 2:** Summary of likelihood ratio χ^2^ from a generalized linear model with a log link and a Poisson residual (analysis of fish counts at tagging; 216 dpf), and a logit link and a binomial residual (analysis of sex ratio at slaughter; *ca*. 1 year old) in (a) a factorial controlled mating design with five paternal (sire) and two maternal (dam) lines of sea bass either considered individually, or (b) grouped by ancestral Atlantic or Mediterranean origin. The nature of genetic effects (additive or nonadditive) is indicated. *df*: degree of freedom

Trait	Fish counts (survival)			Sex ratio		
(a) Five sire lines, two dam lines	*df*	χ^2^	Pr > χ^2^	*df*	χ^2^	Pr > χ^2^
Location	1	1,842	<.0001	1	3.01	0.08
Paternal line (sire; additive)	4	1,482	<.0001	4	13.98	0.007
Maternal line (dam; additive)	1	173.6	<.0001	1	4.88	0.03
Paternal × maternal line (nonadditive)	4	553.1	<.0001	4	11.94	0.02
Residual (goodness of fit)	9	12.92	0.17	9	14.76	0.10

(b) Two sire origins, two dam origins	*df*	χ^2^	Pr > χ^2^	*df*	χ^2^	Pr > χ^2^
Location	1	1,842	<.0001	1	2.73	0.10
Paternal origin (sire; additive)	1	85.8	<.0001	1	0.50	0.44
Maternal origin (dam; additive)	1	248.9	<.0001	1	2.43	0.12
Paternal × maternal origin (nonadditive)	1	457.3	<.0001	1	7.38	0.006
Residual (goodness of fit)	3	4.96	0.17	3	1.20	0.75

Impacts of the fertilization and/or the standardization procedure were also evaluated. The number of fish per cross at tagging was plotted against the percentage of floating (good quality) eggs at 48 hr (Fig. S2). No significant relationship was demonstrated, showing that fertilization/hatching rate was not linked to survival during larval rearing. The number of fish per cross was plotted against the order of manipulation during the equalization of egg numbers per cross at 48 hr, with the idea that eggs manipulated later during the experiment may have suffered from unfavorable holding conditions that may have impacted their early survival. Here again, no significant relationship was demonstrated (Fig. S3). Therefore, survival during larval rearing was found to be highly variable among sea bass origins and revealed a significant nonadditive genetic component. Finally, a strong unbalanced representation of progenies was also demonstrated when considering available reciprocal crosses between ♂_NAT_ × ♀_WEM_ and ♂_WEM_ × ♀_NAT_. When used as sire, NAT was found to produce more offspring (*n*_*off*_ = 1,660; Figure 2) and families (*n*_fam_ = 242 of 255 possible; 95%; Fig. S4) with WEM, than in the reciprocal cross with WEM sires and NAT dams (*n*_*off*_ = 414; *n*_fam_ = 100 of 135; 74%). The difference is significant for the number of families produced (χ^2^ = 9.737, 1 *df*,* p *<* *.002).

**Figure 3:**
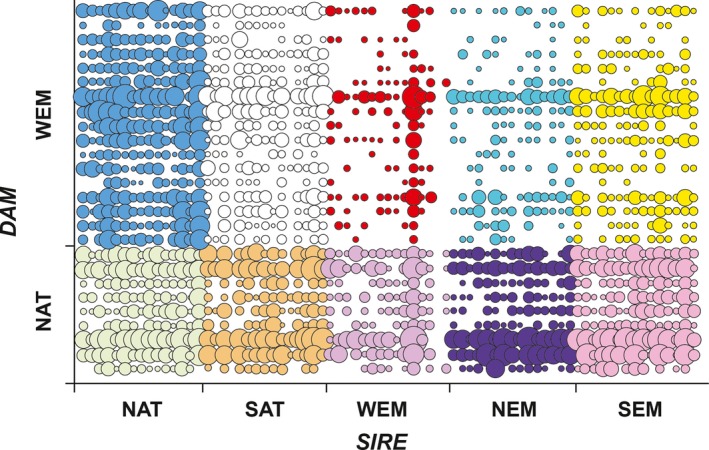
Representation of the number of full‐sib families of sea bass produced by each of the 10 crosses considered in this study. Labels as in Figure 1

The representation of the different sires and dams was quite unbalanced within each line cross, where both sire and dam effects were highly significant in all 10 line crosses (*p *<* *.001, details not reported). The number of progeny per full‐sib family is reported in Figure 3. It clearly demonstrated that sire × dam crosses among Mediterranean populations produced less progenies, together with over representation of particular sires and dams in progenies, especially for WEM (Figure 3 and Fig. S4). Unless there would have been very high variation in fertilization/hatching rate between them, the most likely explanation of such unbalanced patterns remains differential larval survival, both at the parental population level and at the individual sire or dam level within population. This was confirmed when studying the within sire population regression between number of progeny per sire and mean size of sire progeny at tagging. Regression showed a significantly positive relationship between number and individual body mass of the progenies, except in WEM and NAT sires for which the relationship was found positive, but not significant (Fig. S5). A significant relationship was expected from size‐dependent larval survival, but not from differential fertilization rates. As growth could not be directly monitored in early rearing (no individual tagging; necessity to avoid for handling‐induced mortality at early stage), such a result indicated growth potential certainly impacted survival in most sires.

### 3.4 Sex ratio

Proportions of females in each initial cross (N = 10 crosses) are reported in Fig. S6. The mean proportion of females was low (9.4%) in the 2,877 sexed juvenile sea bass with single parentage, and no difference between pregrowing locations was found (χ^2^ = 2.81, 1 *df*,* p *=* *.09). Low proportions of female are the rule in aquaculture conditions (e.g., Piferrer, Blázquez, Navarro, & González, 2005). Female proportions were found to vary according to paternal line (*p *<* *.01), maternal line (*p *<* *.05), and paternal line × maternal line interaction (*p *<* *.05) when both pregrowing locations were combined (Table 2a).

**Figure 4:**
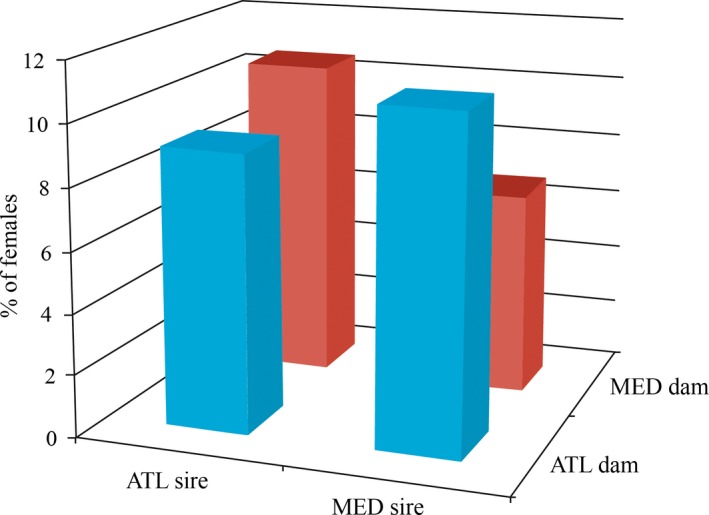
Sex ratio as measured by the proportion of females in each of the parental and reciprocal crosses between NAT and WEM populations. The proportions of females in the 10 sire × dam crosses surveyed in this study are reported in Fig. S6. Labels as in Figure 1

When we grouped the sire and dam populations by origin (Atlantic [ATL] vs Mediterranean [MED]), significantly higher proportions of females were detected for crosses that carried different genomes, i.e., the paternal origin × maternal origin interaction was found significant (*p *<* *.01; Figure 4). This indicated that taking into account the Eastern Mediterranean populations (i.e., the other ancestral population) in MED and comparing them with the ancestral ATL population (NAT and SAT are not genetically distinct and merging data did not affect result) increased the significance of nonadditive effects compared to the original data considering the 10 crosses (Table 2a,b). This result looked like one heterotic effect with reciprocal ATL × MED crosses producing more females compared to “parental” ones. However, because of the incomplete factorial design used in this study, we cannot unambiguously assign this result to heterosis *sensu stricto* (see “Discussion” section for further comments).

We further analyzed the relationship between the proportion of females within sire and the mean body mass of progeny of each sire at tagging in order to test whether body mass at tagging could significantly predict family sex ratio. This relationship was not significant for both the 10 crosses and when grouping sires and dams by their oceanic origin (*p *>* *.25; details not reported).

Finally, a significant relationship was found between the log‐transformed number of fish that survived at 6 months and the proportion of females observed at ~1 year (Likelihood ratio χ^2^ test: χ^2^ = 13.16, 1 *df*,* p *=* *.003). Namely, when one sire line showed higher survival of progenies with one dam line compared to the other, then this sire line also produced more females with this dam line, indicating pleiotropic effects between traits.

### 3.5 Relationship between nonadditive effects and genetic differentiation

**Figure 5:**
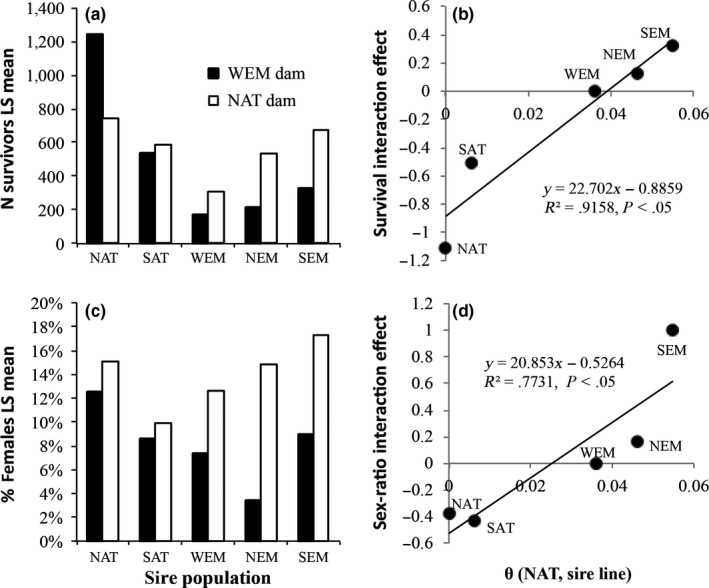
Least square (LS) means of number of survivors (a) and proportion of females (c) for each of the 10 line crosses tested. Linear relationships between the maternal (dam) population × paternal (sire) population interaction effect in GLMs and the estimated pairwise genetic differentiation (θ; *x*‐axis) between the NAT and the five sire populations (NAT, SAT, WEM, NEM, and SEM) are reported for survival (b) and sex ratio (d). The interaction component was set to zero for the WEM × WEM cross. Labels of sires corresponding to each interaction component are indicated on panels b and d. Values of pairwise estimates of genetic differentiation are reported in Table S1

Least square means of survival and sex ratio in each of the 10 population crosses clearly show an increasing advantage of crosses involving NAT dams relative to crosses involving WEM dams when the sires used were more distant to NAT (Figure 5a,c). This was highlighted by the significant correlation of the interaction term between paternal and maternal lines with the genetic distance between NAT and the paternal lines (Figure 5b,d). This pattern was found true for both survival and sex ratio.

This pattern was consistent with the fact that nonadditive effects increased with genetic distance between lines. However, as we did not consider the full diallel between the five populations, we cannot rigorously assess the true metapopulation average and then evaluate the overall nonadditive component of genetic variation at the scale of the whole sea bass metapopulation.

## 4 Discussion

The aim of this study is to provide a fair view of how neutral genetic variation and quantitative variation in two important phenotypes, survival and sex ratio, covary or not at the level of the sea bass metapopulation in the Atlantic and the Mediterranean Sea. It is related to phenogeography, i.e., the study of the distribution and genetic basis of phenotypic trait variation in nature. This study complements the numerous studies from Benharrat, Pasteur, Siau, and Bouain (1983) to Souche et al. (2015) that aimed to explore the distribution‐wide genetic structure of sea bass using molecular markers. In comparison, large‐scale geographic variation of phenotypes was poorly achieved in sea bass and primarily targeted aquaculture traits (Gorshkov et al., 2004; Vandeputte et al., 2014). Implementing an incomplete factorial diallel cross, we were able to estimate and to further explore additive and nonadditive genetic effects in outbred lines directly derived from wild sea bass. The respective roles of additive and nonadditive genetic effects to explain phenotypic variation has gained recent interest in fishes, but they remain largely unexplored (e.g., Gao & Munch, 2013; Houde, Black, Wilson, Pitcher, & Neff, 2015; Neff, Garner, & Pitcher, 2011; Rudolfsen, Figenschou, Folstad, Nordeide, & Soreng, 2005).

### 4.1 Additive and nonadditive genetic effects

Results hereby demonstrated that genetic variation for survival and sex ratio existed in sea bass over the range of populations and F_1_ crosses investigated in this study, with WEM reporting the poorest survival (in aquaculture conditions). The sire population appeared important to survival in sea bass, supporting recent evidence for a paternal role in increased survival of young fish (Álvarez & Garcia‐Vazquez, 2011; Bang, Grønkjaer, Clemmesen, & Høie, 2006; Rideout, Trippel, & Litvak, 2004), but also other broadcast marine spawners (*Styela plicata*; Crean, Dwyer, & Marshall, 2013). A paternal influence was formerly shown for female hatching rate in sea bass, but not for survival (Ky et al., 2012; Saillant et al., 2001). Dedicated experiments would be necessary to precisely investigate whether male influence on hatching rate and early survival correlate in sea bass.

The incomplete diallel design, constrained by logistic reasons as previously explained, is a serious limitation of the present study. Indeed, the additive effects reported here could also contain nonadditive effects, and this especially concerns the sire population effects from populations used only as sires (SAT, NEM, SEM). If this was the case, this could also lead to underestimation of the nonadditive effects, which were significant on sex ratio and survival, but with apparent lower influence than sire “additive” effects. Despite this limitation, results showed that survival and sex ratio were prone to exhibit nonadditive components of genetic variance, which were not found in previously investigated traits such as, e.g., carcass or fillet yields (Vandeputte et al., 2014), but also standard length, body mass, and body condition (M. Vandeputte, B. Guinand, M. Dupont‐Nivet, A. Vergnet, H. Chavanne, P. Haffray, B. Chatain, *unpubl. results*). These results are consistent with reports that pure fitness traits such as survival showing fitness traits more prone to exhibit nonadditive components than traits indirectly related to fitness (Merilä & Sheldon, 1999; Roff & Emerson, 2006).

In the sea bass, it was previously shown that sex ratio was mostly additive at the family level in a North Atlantic population and could be considered a polygenic threshold trait (Vandeputte et al., 2007). Theory predicts that polygenic sex ratio is evolutionarily unstable (Bulmer & Bull, 1982; Van Dooren & Leimar, 2003) and should evolve toward either genetic sex determination with major sex factors or environmental sex determination. This evolution should, however, be highly dependent on environmental and developmental conditions (Van Dooren & Leimar, 2003; Van Doorn, 2014). Hence, it is likely that sex determination could take different directions in different populations and that the main sex determining loci may be distinct from one population to the other (see, e.g., Alexander, Richardson, Edmands, & Anholt, 2015 and references therein). The nonadditive component observed here could be supportive of synergistic effects of different sex factors developed in different populations from a common ancestral polygenic system. This should be further investigated by searching for sex‐linked quantitative trait loci (QTL) in different populations and testing their single and combined effects in specific crosses. First results for the WEM population showed that at least three sex‐ratio QTLs could be detected in sea bass (Palaiokostas et al., 2015), but comparisons with other populations have not been performed yet.

### 4.2 Pleiotropy between survival and sex ratio

The patterns of observed phenotypic values and nonadditive effects for survival and sex ratio were found rather similar in this study. Together with correlations between early survival, early growth, and observed proportions of females, this reflects a pleiotropic effect among traits, a pattern commonly assessed in cultured plants (e.g., Frascaroli et al., 2007; Fu et al., 2009). A positive genetic correlation between growth rate and a trend in expressing the female sex has been demonstrated earlier for the NAT population (Vandeputte et al., 2007), also supporting a pleiotropic effect on sex and growth in sea bass.

### 4.3 A signature of heterosis?

Nonadditive genetic effects on trait performance were detected for both sex ratio as measured by the proportion of females, and survival among the formerly allopatric and ancestral Atlantic and Eastern Mediterranean populations of sea bass. Nonadditive genetic effects were especially found proportional to the observed genetic distance between NAT and the paternal lines, with large positive nonadditive effects found among distant populations (e.g., NAT × NEM, NAT × SEM)). When populations were grouped by oceanic origin (ATL, MED), larger proportions of females and higher survival were observed in crosses involving both Atlantic and Mediterranean individuals compared to crosses of individuals with the same oceanic origin. Note, however, that this pattern is not found when Mediterranean populations remained ungrouped. For instance, the reciprocal NAT × WEM crosses did not report systematic increase in performance compared to their respective parental crosses. This particular case is further discussed below, but results proved that adding the Eastern Mediterranean populations in the analysis to more clearly oppose the two ancestral genomic backgrounds increased the significance of the nonadditive genetic effect. If we cannot rigorously interpret these patterns as heterosis *sensu stricto* because a full diallel factorial cross involving all reciprocal crosses was not performed in our study, results strongly suggest it could be present in sea bass. Indeed, because of the large mutational load classically found in marine organisms (Bierne, Launey, Naciri‐Graven, & Bonhomme, 1998; Launey & Hedgecock, 2001; reviewed in Plough, 2016), heterosis could easily have emerged in sea bass through silencing of deleterious recessive mutations that segregated within each genomic background as expected under the classical theory of dominance. Geographic patterns of nonadditive variation also mirror the classical relationship observed for heterosis. Namely, at low genetic distance mostly occurring *within* population, inbreeding depression prevails because of genetic load (Charlesworth & Charlesworth, 1999), while increased genetic distance *among* populations should promote heterosis (Edmands, 1999; Waser, 1993). We hereby observed low nonadditive genetic effects for populations sharing a common genomic background (NAT × NAT or NAT × SAT), and possibly indicative of inbreeding, while high nonadditive genetic effects were observed among crosses involving different ancestral populations and may be indicative of a heterotic effect. This would have to be investigated further by including at least a SEM dam origin (i.e., the most likely Eastern Mediterranean ancestral population). However, sea bass would could be a rare example of heterosis for a marine fish as—to our knowledge—heterosis was suggested only in hybrids of coastal and Arctic cod (*Gadus morhua*) (Bangera, Ørdegård, Praeble, Mortensen, & Nielsen, 2011), while many cases were reported for marine invertebrates (Bierne, David, Boudry, & Bonhomme, 2002; Edmands, 1999; Pereira, Barreto, & Burton, 2014; Wang & Cote, 2012).

The observation of positive nonadditive genetic effects among Western and Eastern Mediterranean populations of sea bass may also have practical implications. Indeed, population genetics studies have showed that cultured WEM fish escaped from farms in Greece impacted local Eastern Mediterranean populations in a relatively enclosed natural environment (Gulf of Patras; Greece) (Bahri‐Sfar et al., 2005), and in open waters around Cyprus (Brown, Miltiadou, & Tsigenopoulos, 2015). The capacity of foreign progeny to outcompete local individuals could be partly mediated by nonadditive effect for survival (Hänfling, 2007). This was not documented so far in marine fishes, but ecological success of interpopulation hybrids mediated by positive nonadditive genetic effects have been reported for, e.g., freshwater gastropods (Facon, Jarne, Pointier, & David, 2005) and amphibians (Fitzpatrick & Shaffer, 2007). Hence, evidence of positive nonadditive genetic effects in a marine cultured fish deserves further attention as they not only impact production, but may have unforeseen consequences on wild fish. However, the nonadditive effects we observed were on hatchery survival, and it is not straightforward that they would be similar in the wild.

### 4.4 Maladaptation in the WEM × WEM cross

F_1_ individuals from the WEM × WEM cross showed low performance for survival and other traits (e.g., growth, Vandeputte et al., 2014), suggesting possible maladaptation. We previously mentioned the large nuclear gene flow of Atlantic alleles into the WEM population (Tine et al., 2014), while WEM carries only Mediterranean mtDNA haplotypes. Therefore, considering the WEM × WEM cross as a “purebred” is misleading. Quéré et al. (2012) suggested that WEM was possibly a “hybrid swarm.” One of those features is the observation of increased fitness that should allow for ecological success to translate into evolutionary success (see, e.g., Pereira et al., 2014 for a marine example)—which could reflect adaptive introgression promoting the maintenance of hybrids (Hedrick, 2013; Martin, Bouck, & Arnold, 2006). Patterns of survival of the WEM × WEM cross go against high fitness of these “hybrids.” Adaptive introgression patterns or “swarming” at the center of the distribution of parental forms seems relatively common in other fish species (e.g., Machado‐Schiaffino, Juanes, & Garcia‐Vazquez, 2010; Roques, Sévigny, & Bernatchez, 2001; Sinama et al., 2013; Stemshorn, Reed, Nolte, & Tautz, 2011; Walters et al., 2008). However, while those studies described hybridization patterns using molecular markers, we are not aware of studies that reported fitness differences as carried out for WEM in the present study.

It remains that the poor performance of WEM individuals cannot easily be explained. They may have lowered fitness because they do not support aquaculture conditions that were performed outside the Western Mediterranean range during this experiment (Israel and the Azores). This is probably unlikely because lower performance for distinct sets of traits was also observed when WEM fish were reared locally (B. Chatain, pers. obs.) and G×E interactions were not significant in this study. WEM individuals may also have poor fitness because they inherited and still not purged the deleterious alleles initially present in each parental population. This seems theoretically unlikely as recombination and segregation should bring together favorable alleles within the same individuals (and unfavorable alleles in others) and hence improve the efficiency of natural selection by reducing the load (e.g., Charlesworth, 1990; Crow, 1970). However, hybridizing tiger salamanders (*Ambystoma sp*.) demonstrated no recovery of fitness in hybrid populations (Johnson, Fitzpatrick, & Shaffer, 2010), indicating that multigenerational effects of hybridization are complex because hybridizing genomes may become “stabilized” at far lower rates than previously expected (Buerkle & Rieseberg, 2008).

A better understanding of low performance of the WEM × WEM cross may also have strong practical implications as cultured fish from WEM parents represent the main source of seed hatchery production within the Mediterranean Sea and in the Atlantic (European Commission, 2004). Intuitively, the use of this population as a broodstock should be avoided. However, deciding which fish to use or not as broodstock is not economically trivial and also depends on the state of breeding programs. Indeed, for other production traits (e.g., body mass, muscle fat content, processing yields), it has been shown that potential gains produced by one or two generations of artificial selection applied to WEM could easily compensate most of the differences in performance formerly existing among this population and the others (Vandeputte et al., 2014). It is well recognized that recombinant parental genotypes classically have lower fitness, because only specific combinations of parental genomic contributions are likely to produce viable hybrid individuals able to accommodate all the potential constraints arising from intragenomic conflicts (Barton, 2001; Edmands, 2007; Eroukhmanoff, Bailey, & Sætre, 2013). Sea bass data provided in this study suggest that such interindividual variance for survival may occur. Indeed, the performance of the WEM × WEM cross is in average lower than for other crosses, but some sire or dam individuals performed well in terms of fitness in this cross (Fig. S4). The study of genomic variation in introgression rate (Roux et al., 2014) in wild WEM individuals and its consequences on offspring survival could provide better understanding of the low average performance, but high interindividual performances in the WEM × WEM cross.

### 4.5 Darwin's corollary in sea bass

The performance of the two reciprocal crosses between ♂_NAT_ × ♀_WEM_ and ♂_WEM_ × ♀_NAT_ deserve further attention. In these crosses, parents are located on each side of the main sea bass hybrid zone at the Almeria‐Oran front and they do not share the same mtDNA lineage (Coscia et al., 2012; Lemaire et al., 2005). Indeed, regarding survival and female sex ratio, the reciprocal hybrid crosses available in this study did not outperform the two crosses involving parental forms, with hybrid_(♂NAT × ♀WEM)_ > parental_(♂NAT × ♀NAT)_ > hybrid_(♂WEM × ♀NAT)_ > parental_(♂WEM × ♀WEM)_. Ky et al. (2012) also reported that hybrid_(♂NAT × ♀WEM)_ outperformed parental_(♂NAT × ♀NAT)_ for hatching rate. Asymmetric performances of F_1_ crosses are commonly reported in a wide range of organisms even though—contrary to sea bass—F_1_s rarely outcompete their parents as observed in, e.g., numerous freshwater fish species (Bolnick, Turelli, Lopez‐Fernández, Wainwright, & Near, 2008; López‐Fernández & Bolnick, 2007; Russell & Magurran, 2006; Schrader, Fuller, & Travis, 2013).

The observation of such asymmetric barriers to reproductive isolation has been coined as the “Darwin's corollary to Haldane's rule” (Turelli & Moyle, 2007). It reflects the likely presence of asymmetric Dobzhansky–Muller incompatibilities that differentially accumulated depending on time spent since populations diverged (Coyne & Orr, 2004; Turelli & Moyle, 2007). Interpreting results from crosses of a large number of Centrarchid species (i.e., sunfish) with various levels of divergence, Bolnick and Near (2005) showed that when fitness differences occurred among recently “young” species pairs they studied (mtDNA divergence: <6 Myr), they were effectively asymmetric and only found in a single reciprocal cross. Bolnick and Near (2005) showed this asymmetric pattern correlated with the “lag phase” observed before incompatibilities classically accumulate then fix within each taxon during the early stages of speciation (i.e., the “polymorphic prelude” to incompabilities; Cutter, 2012). The 3.8‐Myr divergence estimated between the two monophyletic mtDNA lineages of sea bass (Tine et al., 2014) fits well with this framework. Performances of F_2_ or backcrosses have not been determined in sea bass yet and certainly needs further investigation to gain insight on the basis and strength of reproductive isolation.

### 4.6 Relative performances of reciprocal NAT × WEM crosses

In the present study, the superior performance of the ♂_NAT_ × ♀_WEM_ hybrid cross for early survival and production of females compared to the reciprocal ♂_WEM_ × ♀_NAT_ cross is interesting as it correlates with the relative success of these genotypes in the wild. Indeed, Atlantic mtDNA haplotypes of sea bass have not been observed to naturally introgress the Western Mediterranean Sea population so far, while the reverse was regularly observed (Coscia et al., 2012; Lemaire et al., 2005; Rondon, 2011). This may represent a case of asymmetric selection against migrants resulting in asymmetric reproductive isolation (Gagnaire, Normandeau, & Bernatchez, 2012; Räsänen & Hendry, 2014). Because of pleiotropy (see above), asymmetric selection could act on early survival and favor sex determination of the individuals ♂_WEM_ × ♀_NAT_ cross as males as suggested by Lemaire et al. (2005). Situations in which polygenic sex determination mediated by mtDNA effects could generate striking changes in sex ratio among distinct hybrid crosses have just begun to be explored in *T. californicus* (Alexander et al., 2015; Foley, Rose, Rundle, Leong, & Edmands, 2013) and in freshwater fish species (*Cottus sp*.; Cheng, Czypionka, & Nolte, 2013). Sea bass may represent an interesting fish species to investigate such issues in marine organisms.

## 5 Conclusion

Using individuals derived from wild populations and covering major genetic subdivisions currently recognized for the European sea bass, we performed a phenogeographic study of several phenotypic traits that complements previous phylogeographic studies performed in this species. We showed that both significant additive variance and nonadditive genetic variance were present for early survival and sex ratio. Performance for early survival and sex ratio as measured by the proportion of females may reflect a pleiotropic effect. While potentially suffering from incomplete interpopulation crosses, the expression of nonadditive effects was found related to levels of population divergence and could be indicative of heterosis at the metapopulation level. The relative survival performance of available reciprocal crosses showed correlations with genetic structure observed in the wild, but also highlighted low performance of WEM, the main source of seed hatchery production in sea bass. Genomic resources that accumulated in sea bass should now contribute to a more detailed crosstalk between the genotypic and phenotypic levels of variation to better understand the underpinnings of molecular variation governing expression of traits and DM incompatibilities in the wild or in aquaculture programs. Because the sea bass is only a representative of many marine species studied in this area (Patarnello et al., 2007), we hope that future studies would now more systematically screen variation in relevant phenotypic and fitness traits of marine organisms to complement phylogeography by phenogeography (Conover et al., 2006).

## Conflict of interest

None declared.

## Data accessibility

Data of this study are deposited in Dryad under accession: doi:10.5061/dryad.8r0q8.
