# Supplementary material for: Metapopulation patterns of additive and nonadditive genetic variance in the sea bass (Dicentrarchus labrax)
Source: Ecol Evol. 2017 Mar 21;7(8):2777–90. doi: 10.1002/ece3.2832 (PMC5395432; doi:10.1002/ece3.2832)
Supplement: Supplementary file 1 [file ECE3-7-2777-s001.doc]

**SUPPLEMENTARY INFORMATION**

**for**

**Metapopulation Patterns of Additive and Non-Additive Genetic Variance in the Sea Bass (*Dicentrarchus labrax*)**

B. Guinand, M. Vandeputte, M. Dupont-Nivet, A. Vergnet, P. Haffray, H. Chavanne & B. Chatain

**_____________________________**

**Supplementary Table S1:** Pairwise *F*_ST_ (; *sensu* Weir & Cockerham, 1984) values based on seven microsatellite loci found among the wild samples of sea bass considered in this study (i.e. breeders that initiated the factorial diallel crosses; *N* = 101 correctly amplified individuals). The samples considered in this study represent the currently recognized subpopulations of sea bass (WEM: western Mediterranean [*n* = 32], SAT: southern Atlantic [*n* = 15], SEM: south eastern Mediterranean [*n* = 15], NEM: North Eastern Mediterranean [*n* = 15], NAT: northern Atlantic [*n* = 24]). Results agreed with previous studies that demonstrated significant nuclear genetic differentiation among the Mediterranean and Atlantic subpopulations (e.g., Quéré *et al*., 2012 and references therein), but genetic homogeneity between the NAT and SAT subpopulations (e.g., Fritsch *et al*., 2007). A Bonferroni correction for multiple tests was applied. See details in the text.

|  | **SEM** | **WEM** | **SAT** | **NAT** |
| --- | --- | --- | --- | --- |
|  |  |  |  |  |
| **NEM** | 0.0340*** | 0.0236*** | 0.0590*** | 0.0548*** |
| **SEM** |  | 0.0465*** | 0.0609*** | 0.0463*** |
| **WEM** |  |  | 0.0293*** | 0.0361*** |
| **SAT** |  |  |  | 0.0062^NS^ |

***: P < 0.001; NS: not significant

**Associated references:**

Fritsch, M., Morizur, Y., Lambert, E., Bonhomme, F. &. Guinand, B. 2007 Assessment of sea bass (*Dicentrarchus labrax*, L.) stock delimitation in the Bay of Biscay and the English Channel based on mark-recapture and genetic data. *Fish. Res.* **83:** 123–132.

Quéré, N., Desmarais, E., Tsigenopoulos, C.S., Belkhir, K., Bonhomme, F. & Guinand, B. 2012. Gene flow at major transitional areas in sea bass (*Dicentrarchus labrax*) and the possible emergence of a hybrid swarm. *Ecol. Evol*. **2:** 3061-3078.

Weir, B.S. & Cockerham, C.C. 1984. Estimating *F*-statistics for the analysis of population structure. *Evolution* **38:** 1358–1370.

**Figure Suppl. S1:** Genetic differentiation among wild sea bass samples illustrated by the positions of male and female wild individual breeders that initiated the crosses considered in this experiment onto the map of a factorial correspondence analysis. Individual genotypes at seven microsatellite loci were used to generate this map. Analysis was performed with Genetix v4.05 (http://kimura.univ-montp2.fr/genetix/). The three first axis of the analysis summarise 83.91% of the total variance of the data set (axis 1: 40.04%; axis 2: 24.57%; axis 3: 19.30%). Details on significance of genetic differentiation for each pairwise population comparisons are reported in Suppl. Table S1. WEM: western Mediterranean, SAT: southern Atlantic, SEM: south eastern Mediterranean, NEM: North Eastern Mediterranean, NAT: northern Atlantic.

**Figure Suppl. S2:** Number of fish per cross at tagging (mean individual weight ~20g; 187 dpf in Israel; 216 dpf in Portugal) in relation to the percentage of floating eggs at 48 hours post-fertilization. The percentage of floating eggs is an indicator of the fertilization rate (Carillo *et al*., 1989). No significant relationship was demonstrated, showing that fertilization/hatching rate is not linked to survival during larval rearing. Significant heterosis detected in the data is then not biased by difference in fertilization or hatching rates.

**Associated reference**:

Carrillo, M., Bromage, N., Zanuy, S., Serrano, R. & Prat, F. 1989. The effect of modifications in photoperiod on spawning time, ovarian development and egg quality in the sea bass (*Dicentrarchus labrax* L.). *Aquaculture* **81:** 351–365.

**Figure Suppl. S3:** Number of fish per cross at tagging in relation to the manipulation order at 48 hours post-fertilization. As the relationship was not found significant, eggs manipulated later during the experiment did not suffered from unfavourable holding conditions that may have substantially impacted their survival. Significant non-additive interaction effect detected in the data is then not biased by manipulation of crosses.

**Fig. Suppl. S4 :** Contribution of sire and dam to the progenies at tagging (216 days post-fertilization)

**A :** Representation of the 73 sires in the progenies at tagging. Two sires over 75 considered in this study have no progeny (SAT#04 and WEM#14). The ordinate axis represents the number of offspring produced by each sire. Labels as in Fig. 1.

**B :** Representation of the 26 dams in the progenies at tagging (~20g). Labels as in Fig. 1.

**Fig. Suppl. S5 :** Relationship between the number of progeny by sire and the mean body mass of progenies at tagging (216 days post-fertilization). Regression lines are reported for each sire’s population, together with significance of the linear relationship. Labels as in Fig. 1.

**Fig. Suppl. S6 :** Sex ratio as measured by the proportion of females in each of the ten sire × dam crosses. Labels as in Fig. 1.
